# Supplementary material for: Unfolding the physics of URu2Si2 through silicon to phosphorus substitution
Source: Nat Commun. 2016 Feb 19;7:10712. doi: 10.1038/ncomms10712 (PMC4762885; doi:10.1038/ncomms10712)
Supplement: Supplementary Information — Supplementary Figures 1-6, Supplementary Table 1, Supplementary Discussion and Supplementary Reference [file ncomms10712-s1.pdf]

I. Supplementary Figures

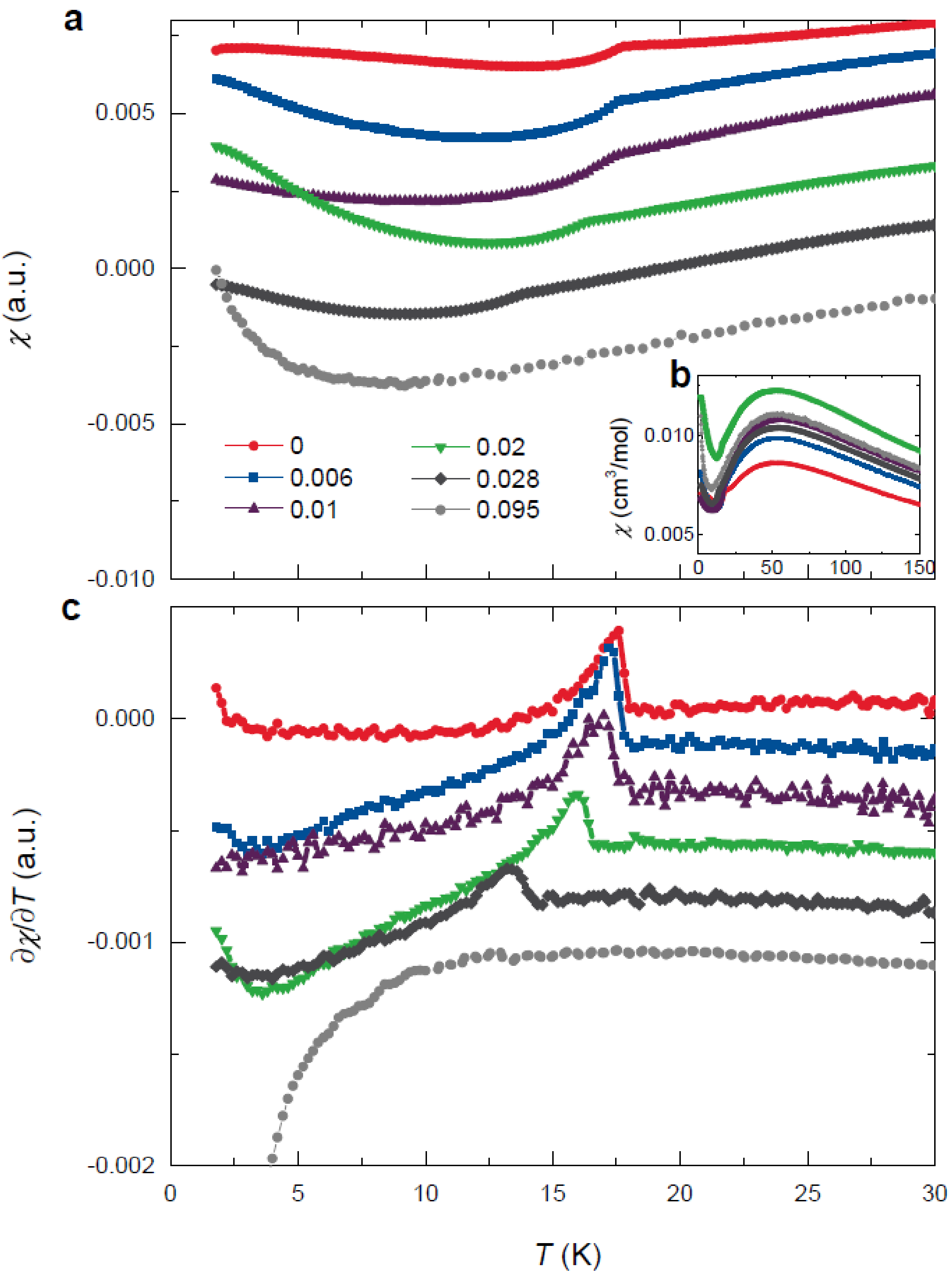

Supplementary Figure 1. Magnetic susceptibility. (a) The low temperature magnetic susceptibility $\chi = M/H$ vs. temperature T for $URu_2Si_{2-x}P_x$ at several x acquired in an applied field of H = 5 kOe. For clarity, the curves are offset by constants. (b) $\chi(T)$ vs. T. (c) Derivative of the magnetic susceptibility $\partial\chi/\partial T$ vs. T for several x.

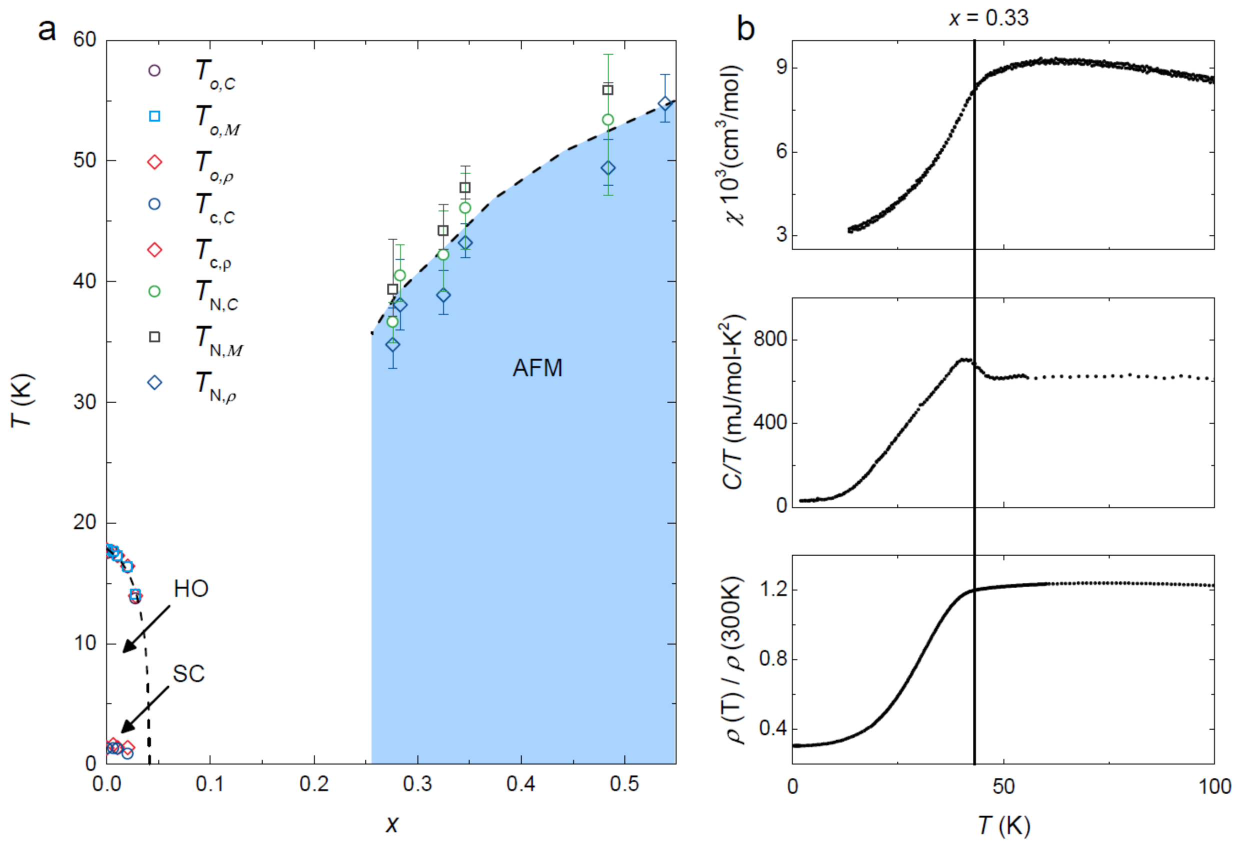

Supplementary Figure 2. Extended phase diagram. (a) Temperature T vs concentration x phase diagram for $URu_2Si_{2-x}P_x$ constructed from magnetic susceptibility χ, resistivity ρ, and specific heat C measurements. (b) Select data for $x = 0.33$ the vertical line defines the antiferromagnetic transition T_N.

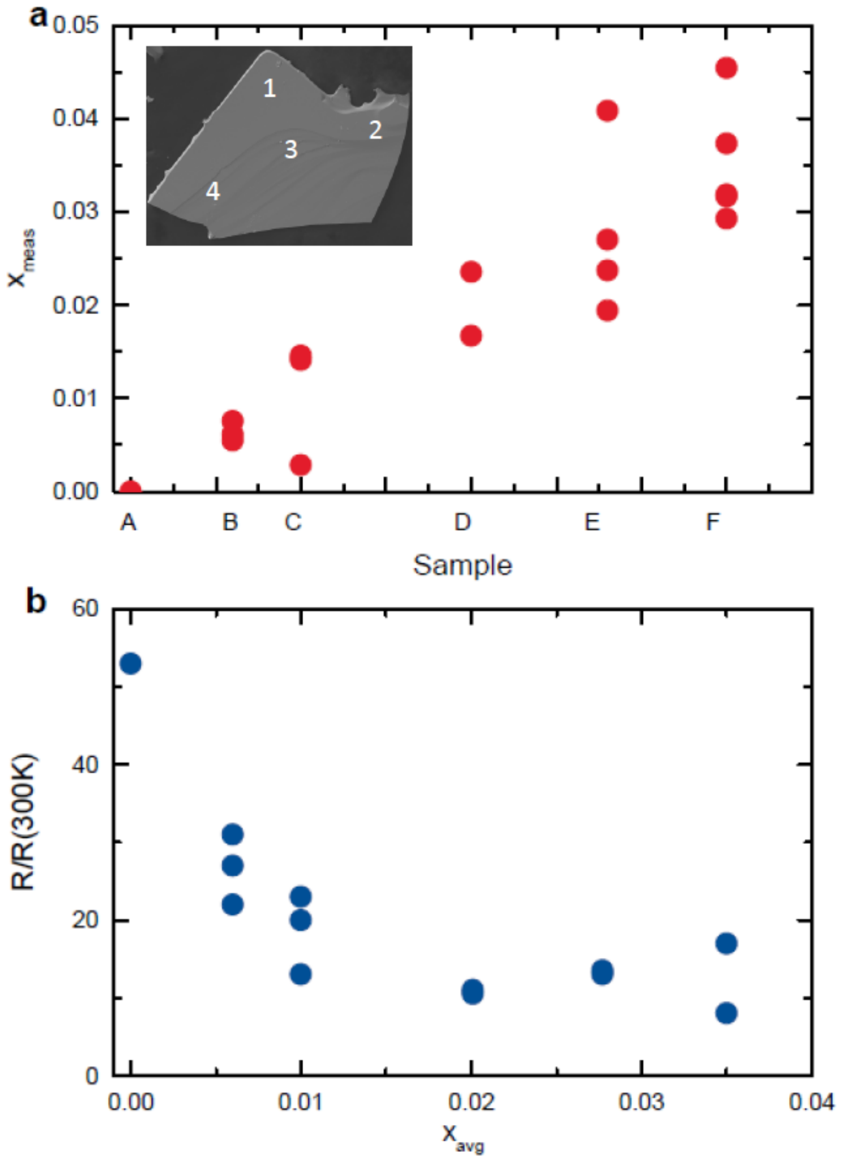

Supplementary Figure 3. Evolution of chemical parameters and residual resistivity ratio. (a) Representative data sets acquired using electron dispersive spectroscopy (EDS) for $URu_2Si_{2-x}P_x$. Samples from distinct batches (labeled A-F) were affixed to conducting carbon tape for measurement. EDS measurements were made on each sample in several different regions, as shown in the inset. The results of the measurements are plotted vs. sample label, where we find a small spread in x_{meas}. Averages from these values are used in the manuscript. (b) The residual resistivity ratio $RRR = R/R(300\ \mathrm{K})$ vs. x_{avg} for $x < 0.04$. Results are shown for several different samples at each x, demonstrating that there is a small amount of batch variation for a given x.

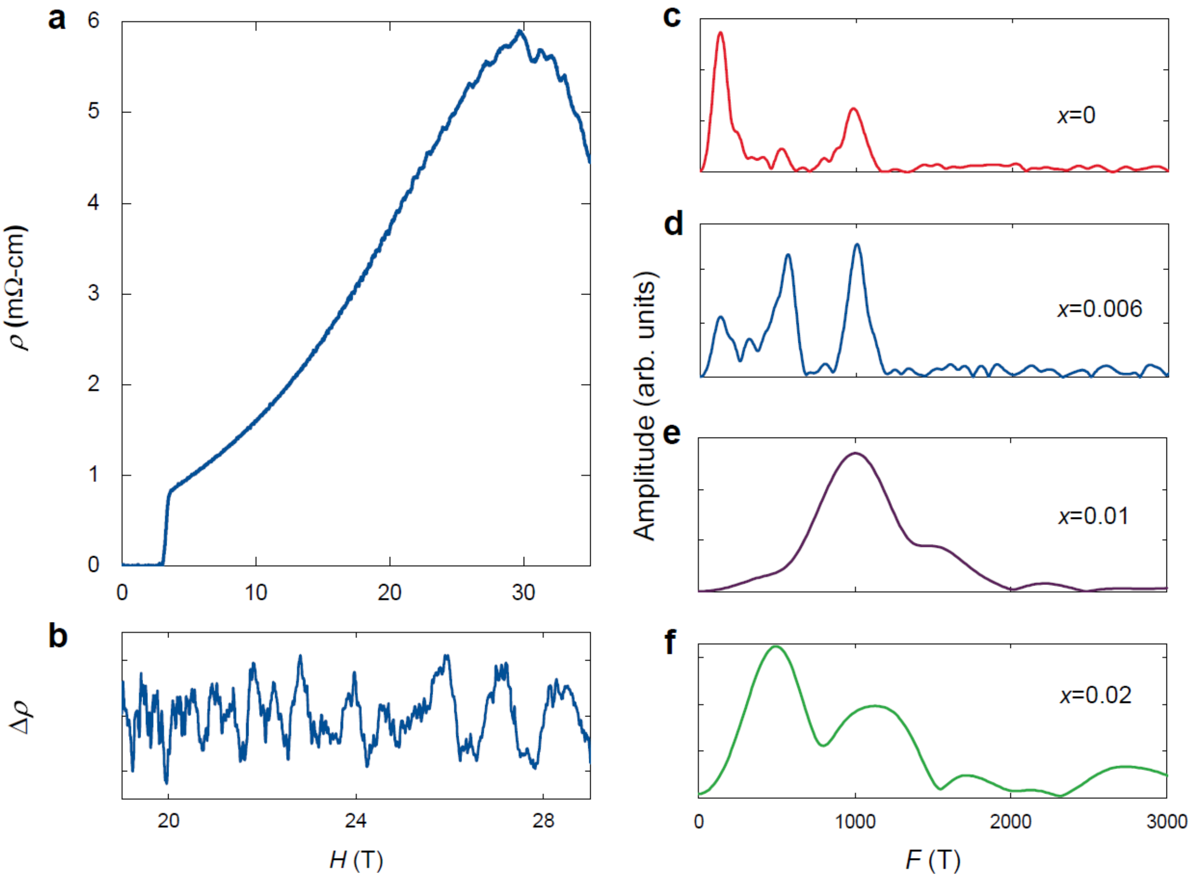

Supplementary Figure 4. Electrical transport under high magnetic fields. (a) Magnetoresistance at 50 mK for x = 0.006 $URu_2Si_{2-x}P_x$ in fields up to 35 T. The raw data clearly shows quantum oscillations, demonstrating the high sample quality. (b) Background subtracted magnetoresistance for x = 0.006. (c-f) Quantum oscillations are observed for samples at x = 0 - 0.02. We show the Fourier spectrum obtained at 50mK for these concentrations.

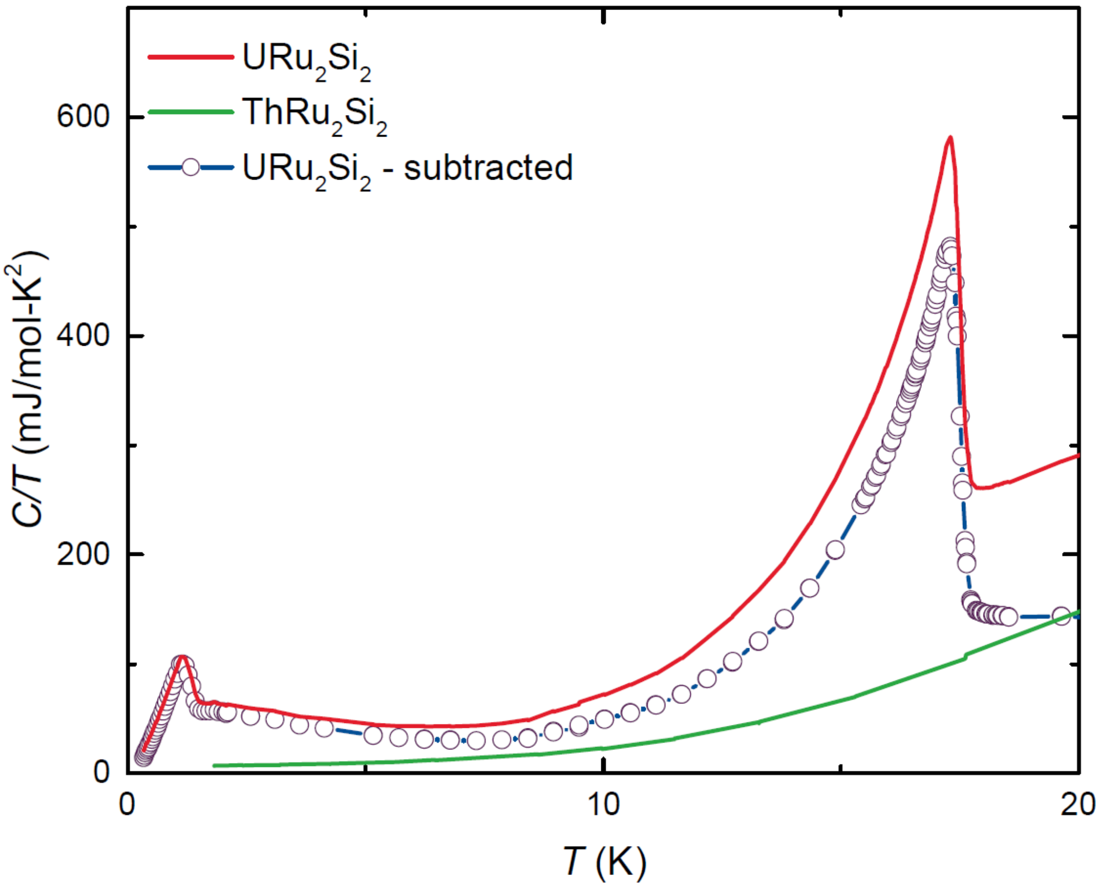

Supplementary Figure 5. Heat capacity analysis. Heat capacity *C* divided by temperature *T* for URu_2Si_2 (red line) and $ThRu_2Si_2$ (green line). Also shown is the 5*f* contribution to the heat capacity of URu_2Si_2 (blue circles) divided by temperature C_{5f}/T, which was acquired as described in the text.

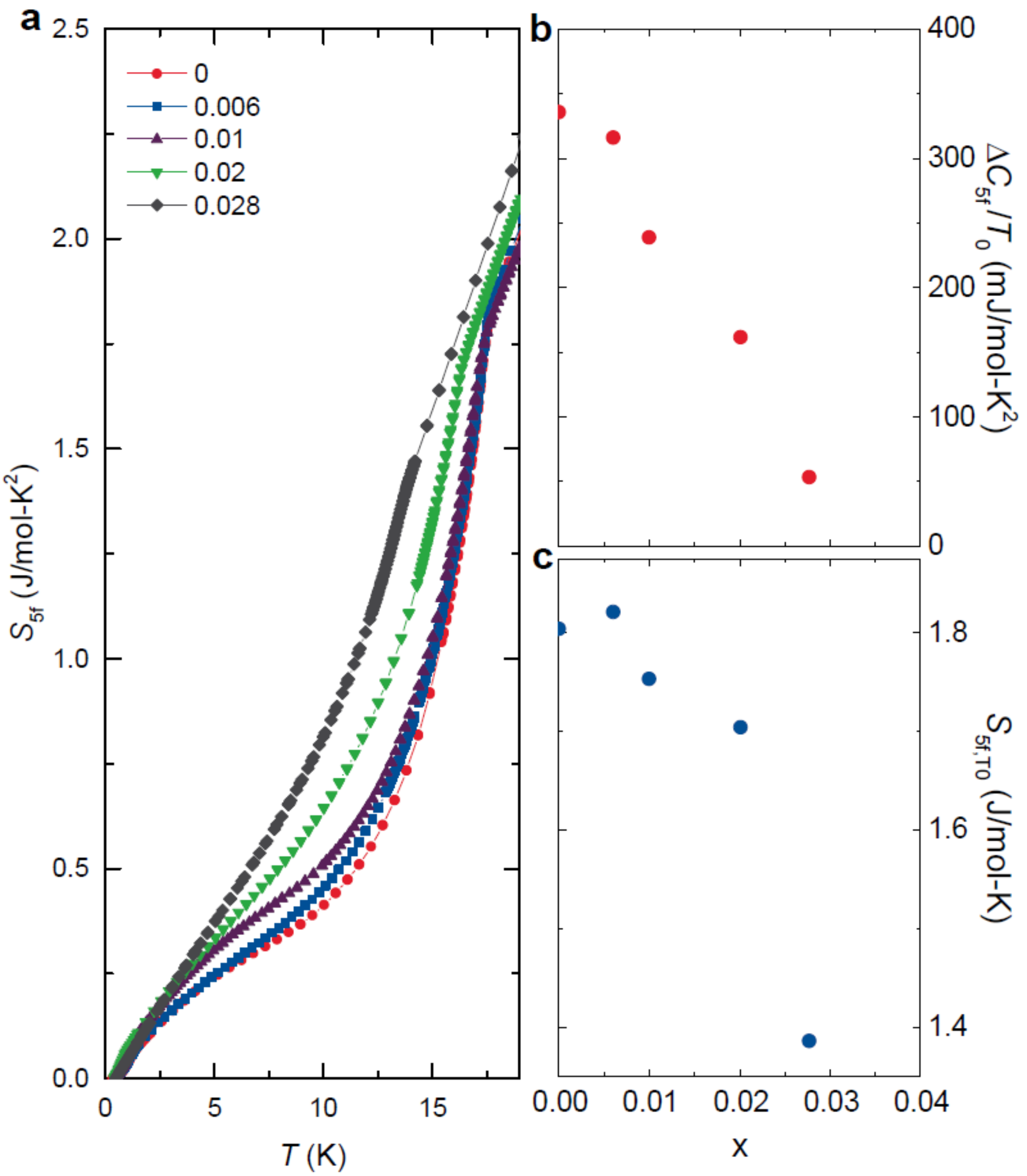

Supplementary Figure 6. 5*f* entropy. (a) The 5*f* contribution to the entropy S_{5f} vs. T for $URu_2Si_{2-x}P_x$. (b) The size of the discontinuity in the heat capacity divided by temperature at the hidden order transition temperature $\Delta C_{5f}/T_0$ vs. phosphorous concentration x. (c) The entropy recovered at the hidden order transition $S_{5f}(T_0)$ vs. x.

II. Supplementary Tables

Sample	x_{avg}	x std. dev.
B	0.006	0.001
C	0.01	0.005
D	0.02	0.003
E	0.028	0.008
F	0.035	0.005

Supplementary Table 1. Chemical Analysis. Results from electron dispersive spectroscopy (EDS) measurements for $URu_2Si_{2-x}P_x$ showing the average x value and its standard deviation, as calculated from the data shown in Supplementary Figure 3.

III. Supplementary Discussion

Magnetic susceptibility. Magnetization $M(T,H)$ measurements were carried out for mosaics of single crystals for temperatures T = 1.8 - 350 K under an applied magnetic field of H = 5 kOe applied parallel to the c-axis using a Quantum Design Magnetic Property Measurement System. Magnetic susceptibility χ is defined as the ratio M/H. The low temperature $\chi(T)$ data for $URu_2Si_{2-x}P_x$ at several x are shown in Supplementary Figure 1a, where the hidden order phase transition T_0 appears as a sharp kink that reduces χ. The evolution of T_0 is illustrated by the derivative of the magnetic susceptibility $\partial\chi/\partial T$ vs. T (Supplementary Figure 1c), where it appears as a lambda-like feature. As discussed in the main text $T_0(x)$ extracted from $\chi(T)$ is in close agreement with results from electrical resistivity and heat capacity.

The extended *T*-*x* phase diagram. Results from magnetic susceptibility χ, resistance R, and specific heat C measurements are collected in Supplementary Figure 2 to construct the T-x phase diagram up to $x \approx 0.55$. Attempts at chemical substitution for $x > 0.55$ were not successful. As discussed in the main manuscript, hidden order and superconductivity are rapidly suppressed and disappear near $x \approx 0.03$. For $0.035 \leq x \leq 0.25$ we find a broad region that exhibits Kondo lattice behavior (see main text) but does not support an ordered ground state. For $x > 0.25$, antiferromagnetism abruptly appears in the correlated electron background, where the Néel temperature increases with x and reaches a maximum near 55 K. Supplementary Figure 2b shows an example of transport and thermodynamic signatures for antiferromagnetism at T_N. This brings into question the nature of the relationship between hidden order and antiferromagnetism.

Determination of phosphorous content. The results of electron dispersive spectroscopy (EDS) measurements on several $URu_2Si_{2-x}P_x$ samples are shown in Supplementary Figure 3a for $x \leq 0.035$. The method in which the data were taken is illustrated in the Supplementary Figure 3a inset, which shows an image of a representative sample that was collected using a Zeiss 1540 EsB scanning electron microscope (SEM). Chemical analysis measurements were made using an EDAX TSL detector on each sample at several different spots, as indicated by the numerical labels. The measured values of x are shown vs. the labels A-F, where the resulting average values x_{avg} are used in the data sets presented throughout the manuscript. The standard deviations for each concentration are collected in Supplementary Table 1. In Supplementary Figure 3b, we show the room temperature normalized electrical resistance RRR =

$R/R(300K)$ vs. x_{avg}, where we find a monotonic decrease in *RRR* with increasing x_{avg} up to 0.02, after which *RRR* becomes roughly constant. This monotonic behavior is consistent with increasing disorder due to phosphorous substitution. Note that $RRR \approx 10$ is comparable to what is normally seen for as-grown single crystals of URu_2Si_2 produced by the Czochralski technique.

Electrical resistivity and measurement of Shubnikov de Haas Oscillations. Zero magnetic field electrical resistance R was measured using the He3 option in Quantum Design Physical Properties Measurement System for temperatures 400 mK < T < 300 K. Several individual crystals were measured for each concentration, which revealed a high degree of batch uniformity. Samples that are produced using our molten metal flux technique are quite small, making it difficult to reliably measure their thicknesses, which are typically between 30 – 80 microns. This leads to some uncertainty in the absolute resistivity values. For this reason, normalized resistivity is presented in the main text. For all of the concentrations reported, the room temperature resistivity is between 300 - 500 μΩcm, consistent with prior observations for URu_2Si_2.[1]

The angular dependence of the superconducting upper critical field was measured using the superconducting magnet (SCM-1) dilution refrigerator system at the National High Magnetic Field Laboratory for H < 18 T and T = 20 mK. Additional magnetoresistance measurements were performed at the National High Magnetic Field Laboratory, Tallahassee, up to magnetic fields of 35 tesla and at T = 50 mK. All high field measurements show quantum oscillations for $x \leq 0.02$ (Supplementary Figure 4) highlighting the high quality of these specimens.

Heat capacity. Heat capacity measurements were performed for mosaics of single crystals using the He3 option in a Quantum Design Physical Properties Measurement System for temperatures 400 mK < T < 20 K. In Supplementary Figure 5 we show heat capacity data for URu_2Si_2 and its nonmagnetic isostructural analogue $ThRu_2Si_2$. In order to obtain the 5*f* contribution to the heat capacity C_{5f}/T for URu_2Si_2, we subtracted C/T for $ThRu_2Si_2$ from that of URu_2Si_2. This method gives a reasonable subtraction of the phonon contribution since $ThRu_2Si_2$ is a typical Pauli paramagnet with no structural phase transition. The quantity that remains after this subtraction is primarily from non-lattice terms, which we label C_{5f}/T. Over the small x range presented here, we assume that the lattice contribution does not change.

The 5*f* contribution to the entropy S_{5f} for $URu_2Si_{2-x}P_x$ was acquired by integrating C_{5f}/T starting from T = 400 mK (Supplementary Figure 6a). While this method slightly underestimates the actual entropy, it gives insight into the trends upon varying x. For instance, the hidden order transition temperature appears as a kink near 17.5 K, for $x = 0$, which is suppressed to lower temperatures with increasing x. The thermodynamic parameters that quantify the hidden order transition, $\Delta C_{5f}/T_0$ and $S_{5f,T0}$, are shown in Supplementary Figure 6b,c. Both of these quantities are monotonically suppressed with increasing x, commensurate with $T_0(x)$. This is in contrast to the trends for $\Delta C_{5f}/T_c$ and $S_{5f,Tc}$which evolve non-monotonically, as discussed in the main text.

II. Supplementary References

[1] Baumbach, R. E., Fisk, Z., Ronning, F., Movshovich, R., Thompson, J. D., and Bauer, E. D., "High purity specimens of URu2Si2 produced by a molten metal flux technique," *Phil. Mag.* **94**, 3663 (2014).
